# Nitrogen addition promotes early-stage and inhibits late-stage decomposition of fine roots in *Pinus massoniana* plantation

**DOI:** 10.3389/fpls.2022.1048153

**Published:** 2022-11-14

**Authors:** Lijun Wang, Yafei Shen, Ruimei Cheng, Wenfa Xiao, Lixiong Zeng, Pengfei Sun, Tian Chen, Meng Zhang

**Affiliations:** ^1^ Key Laboratory of Forest Ecology and Environment, National Forestry and Grassland Administration, Ecology and Nature Conservation Institute, Chinese Academy of Forestry, Beijing, China; ^2^ Co-Innovation Center for Sustainable Forestry in Southern China, Nanjing Forestry University, Nanjing, China

**Keywords:** nitrogen addition, root orders, decomposition, initial chemical quality, lignin degradation

## Abstract

Increasing atmospheric nitrogen (N) deposition has a profound impact on the ecosystem functions and processes. Fine root decomposition is an important pathway for the reentry of nutrients into the soil. However, the effect of N addition on root decomposition and its potential mechanism is not well understood with respect to root branch orders. In this study, we conducted a 30-month decomposition experiment of fine roots under different concentrations of N addition treatments (0, 30, 60, and 90 kg N ha^-1^ year^-1^, respectively) in a typical *Pinus massoniana* plantation in the Three Gorges Reservoir Area of China. In the early stage of decomposition (0−18 months), N addition at all concentrations promoted the decomposition of fine roots, and the average decomposition rates of order 1–2, order 3–4, order 5–6 fine roots were increased by 13.54%, 6.15% and 7.96% respectively. In the late stage of decomposition (18−30 months), high N addition inhibited the decomposition of fine root, and the average decomposition rates of order 1–2, order 3–4, order 5–6 fine roots were decreased by 58.35%, 35.43% and 47.56% respectively. At the same time, N addition promoted the release of lignin, carbon (C), N, and phosphorus (P) in the early-stage, whereas high N addition inhibited the release of lignin, C, N, and the activities of lignin-degrading enzyme (peroxidase and polyphenol oxidase) in the late-stage. The decomposition constant (*k*) was significantly correlated with the initial chemical quality of the fine roots and lignin-degrading enzyme activities. The higher-order (order 3–4 and order 5–6) fine roots decomposed faster than lower-order (order 1–2) fine roots due to higher initial cellulose, starch, sugar, C concentrations and higher C/N, C/P, lignin/N ratios and lower N, P concentrations. In addition, low N (30 kg N ha^-1^ year^-1^) treatments decreased soil organic matter content, whereas high N (90 kg N ha^-1^ year^-1^) treatment had the opposite effect. All the N treatments reduced soil pH and total P content, indicating that increased N deposition may led to soil acidification. Our findings indicated that the effect of N addition on decomposition varied with the decomposition stages. The decomposition difference between the lower-order and higher-order fine roots were controlled strongly by the initial chemical quality of the fine roots. This study provides new insights into understanding and predicting possible changes in plant root decomposition and soil properties in the future atmospheric N deposition increase scenarios.

## Introduction

Over the past century, food and energy production and other human activities have increased the release of reactive nitrogen (N) into the atmosphere by 3–5-fold (IPCC, 2007). China has become the third-largest N depositor globally, with an average annual total deposition of approximately 21.1 kg N ha^-1^ year^-1^, which is expected to increase further (Liu et al., 2013). Increasing atmospheric N deposition has a profound impact on the functions and nutrient cycling processes of ecosystem (Van Groenigen et al., 2017; Liu et al., 2019; Yu et al., 2021; Wu et al., 2022).

Fine root decomposition plays an irreplaceable role in subterranean ecosystems (Dong et al., 2020). Nitrogen, phosphorus (P), and other nutrients released during fine root decomposition affect plant growth and soil biological activity. The organic matter entering the soil through fine root decomposition accounts for 14% ~ 86.8% of the total input (Usman et al., 2000; Parton et al., 2007). The carbon (C) returned to soil from fine roots is 4–5 fold that of from the aboveground litter. Therefore, fine root decomposition is an important pathway for the reentry of nutrients into the soil (Fahey and Arthur, 1994; Steinaker and Wilson, 2005; Xu et al., 2022). It is important to clarify the effects of increased N deposition on fine root decomposition and the ecosystem C balance (Reay et al., 2008; Kou et al., 2015; Sun et al., 2016; Song et al., 2016).

Numerous previous studies on the effect of elevated N deposition on fine root decomposition have made some progress, but the results are controversial (Berg and Matzner, 1997; Kou et al., 2015; Tu et al., 2015; Song et al., 2016; Dong et al., 2020). For example, some studies found that adding N significantly decreased the decomposition rate due to the increase of lignin concentration and the combination of inorganic N ions with acid nonhydrolyzable residues or the change of soil environment (Tu et al., 2014; Kou et al., 2015; Xia et al., 2017). On the contrary, a study in temperate grasslands found that N addition, in either organic or inorganic forms, stimulated the decomposition rate of fine roots (Dong et al., 2020). In addition, Song et al. (2015); Song et al. (2016) suggested that low-level (30 kg N ha^-1^ year^-1^) N addition promoted fine root decomposition rate and nutrient elements release, whereas high N (≥60 kg N ha^-1^ year^-1^) addition had the opposite effects. Other studies found that N addition promoted the early stage of decomposition due to the higher C concentrations and lower N concentrations, while inhibited the later stage of decomposition due to the increase of lignin concentration and the decrease of lignin degrading enzyme activity (Berg and Matzner, 1997; Hobbie et al., 2012). A meta-analysis found that the effect of N addition on litter decomposition depends on the initial quality of substrate, nitrogen deposition rate in environment and the concentration and rate of nitrogen application (Knorr et al., 2005). Overall, the above studies have proposed some hypotheses to explore the potential mechanisms of N addition on fine roots decomposition, but there appears to be mixed and sometimes even conflicting findings.

Nearly half a century ago, most studies considered fine roots a homogeneous system, which holds that the root of all less than 2 mm are basically the same in structure and physiology. However, recent studies have shown that a single diameter class definition ignores the heterogeneity of internal structure and function of the fine root systems (Pregitzer et al., 2002; Guo et al., 2004; Yang et al., 2019). The root system is composed of different branching levels (Guo et al., 2004), and there are significant differences in the physiological processes of different orders fine roots. These differences in root function affect the histochemical composition, including the C/N ratio and concentrations of non-structural carbohydrates, calcium, phenolic compounds, and lignin (Guo et al., 2004; Goebel et al., 2011). Therefore, the differences in chemical composition between different root branch orders may affect their decomposition rate. A few studies on root decomposition have found that the decomposition rate of the lower-order fine roots was slower than that of the higher-order fine roots (Kou et al., 2015; Yang et al., 2019). These studies suggested that the lower-order fine roots are more easily colonized by ectomycorrhizal (EM) mycorrhizal fungi and the chitin rich mycorrhizal sheath may reduce the decomposition of the fine roots (Langley et al., 2003a; Langley and Hungate, 2003b; Guo et al., 2008). Other studies suggested that the slow decomposition of lower-order fine roots is mainly driven by their high content of acid-insoluble substances (Parton et al., 2007; Xiong et al., 2013; Liu et al., 2019), which is unrelated to the initial N concentration (Sun et al., 2013; Xiong et al., 2013). Yang et al. (2019) indicated that the slow decomposition of the lower-order roots is due to the combined effects of higher initial N content and lower C content. However, Shen et al. (2017) has the opposite conclusion that the lower-order fine roots decomposed faster due to the higher C/N ratio and the lower N concentration during the decomposition process and the higher sensitivity to soil temperature, moisture, Ca concentration. The findings regarding fine root decomposition are controversial, and the potential mechanism is unclear; therefore, more experiments are required for better understanding.

As China’s key sensitive ecological area and the ecological barrier between the middle and lower reaches of the Yangtze River, the Three Gorges Reservoir area has an atmospheric N deposition flux of approximately 30 kg N ha^-1^ year^-1^ which is quite high (Zheng et al., 2014). *Pinus massoniana* plantations are widely distributed and contribute substantially to both the economic and ecological value in the Three Gorges Reservoir area of China (Zeng et al., 2018). The effect of increased nitrogen deposition on root decomposition of *Pinus massoniana* is bound to change the soil carbon pools of local forest ecosystems. Therefore, in this study, we conducted the decomposition experiments of three orders fine roots under the condition of different concentration N treatments for 30 months in a typical *Pinus massoniana* plantation. Our aim was to clarify the effect of nitrogen addition on different order fine root decomposition and the reasons for the differences. We proposed the following hypotheses: (1) N addition promotes fine root decomposition in the early decomposition stages but inhibits decomposition in the late stages due to the inhibition of lignin degradation. (2) The higher-order fine roots decompose faster than the lower-order fine roots due to the difference in initial chemical quality.

## Materials and methods

### Site description

The study was conducted in Shuangshan (30° 46′ N, 110° 55′ E) of Zigui County, Hubei Province, China, at an elevation of approximately 825 m. The site has a subtropical continental monsoon climate with an annual average temperature of 14 to 22°C. The average yearly rainfall is 1400 mm, concentrated from April to September. The *Pinus massoniana* plantation was established by air sowing in the 1980s. The average height, diameter at breast height and density of the tree were 16.96 m, 18.3cm, 675 trees ha^-1^, respectively. Yellow-brown and purple are the main soil types, with a depth of approximately 40 cm. The initial soil organic matter content (g/kg) and pH was 22.30 ± 5.9, 4.93 ± 0.06 (mean values ± standard error) at 0−20 cm depth. The nutrient concentration (mg/kg) was 544.03 ± 0.93, 138.68 ± 29.36, 3728.39 ± 89.80, 39.93 ± 0.91, and 68.40 ± 12.78 for total N, P, potassium (K), zinc (Zn), and calcium (Ca), respectively. The main associated shrubs are *Camellia sinensis*, *Eurya nitida, Viburnum erosum*, and herbs are *Dryopteris fuscipes*, *Houttuynia cordata*, *Senecio scandens*.

### Experimental design

In February 2019, based on the local atmospheric N deposition background value (30 kg N ha^-1^ year^-1^), 12 plots (2 m × 2 m) were established at the experimental site with four N treatments: control (CK, 0 kg N ha^-1^ year^-1^), low N (LN, 30 kg N ha^-1^ year^-1^), medium N (MN, 60 kg N ha^-1^ year^-1^), and high N (HN, 90 kg N ha^-1^ year^-1^). Each treatment was replicated three times. The plots were separated by a 10 m wide buffer zone and isolated with polyvinyl chloride plates inserted into 20 cm depth soil. The forms of nitrogen deposition include dry deposition and wet deposition. The dry deposition is mainly composed of gaseous nitrogen containing pollutants, while the wet deposition is mainly composed of granular ammonium salt and nitrate. In China, ammonium deposition accounts for 68% of the total nitrogen deposition and nitrate deposition accounts for 32% (Zheng et al., 2014; Sun et al., 2016). Therefore, ammonium nitrate (NH_4_NO_3_) was selected as the N source in this study. The annual application amount of NH_4_NO_3_ was evenly divided into 12 equal parts. An appropriate amount of NH_4_NO_3_ was dissolved in 2 L of water and sprayed uniformly in the quadrat at the beginning of each month, starting from February 2019. Precisely the same amount of plain water was sprayed in the control group.

### Decomposition and sampling

In early January 2019, taproots were found at the base of the *P. massoniana* trunk (outside the N addition site). We looked for root order branches approximately 2 m outward along the lateral roots on the taproots. Then, the root system was carefully separated from the soil particles, ensuring that the smallest root tip at the root end was intact. According to the method described by Pregitzer et al. (2002), the roots were divided into different branching orders: order 1-2 (the lower-order fine roots), order 3–4 and order 5–6 (were grouped as the higher-order roots).

All the fine roots of the same order class were mixed, and a 1.0 g sample of each root order class was placed in a nylon 150-mesh litterbag (10 cm ×10 cm). In February 2019, litterbags were randomly placed in the soil of each plot with a depth of 10 cm. Five samplings were conducted during the 30-month decomposition in May, August, November (2019), and August (2020 and 2021). A total of 1260 litterbags were collected in this fine root’s decomposition experiment (4 treatments × 3 replicates × 3 root orders × 5 samplings ×7 replicates). On each harvest date, soil samples (at 0−20 cm depth) from the 12 plots and 7 replicate litterbags for each root order were collected and quickly transferred to the laboratory. The roots were carefully separated from the soil particles, dried in an oven (65 °C for 48 h), and weighed to calculate the dry mass. Some soil samples were screened with a 2 mm mesh and placed in a 4 °C refrigerator to measure soil enzyme activity, whereas others were air-dried indoors and passed 2 mm and 0.149 mm mesh for measuring soil pH and the content of soil organic matter, total nitrogen, and total phosphorus (P).

### Chemical analysis

Subsamples of fine roots and soil were analyzed as follows. The concentrations of cellulose, starch and soluble sugar in fine roots were determined by anthrone colorimetry (Ververis et al., 2004; Guo et al., 2022). Lignin concentrations were determined using the acid detergent fiber (ADF) method (Rowland and Roberts, 1994). Total C and organic matter were determined by the potassium dichromate oxidation method (Nelson and Sommers, 1982). Total N, total P and pH-value were determined by the Kjeldahl method, molybdenum antimony colorimetric method and electrode method, respectively (Zhu et al., 2014; Wang et al., 2021). L-3,4-dihydroxyphenylalanine (25000 μmol L^-1^) was used as the substrate to mark the activities of polyphenol oxidase and peroxidase according to Saiya-Cork et al. (2002), and a multifunctional microplate fluorometer (SpectraMax i3x, Molecular Devices, Beckman Coulter, CA, USA) was used to measure the absorbance.

### Statistical analysis

The residual mass rate (MR) of the fine roots at each stage was calculated using equation (1). The percent chemical remaining mass *(E)* was expressed using equation (2) (Fu et al., 2021). An exponential decay model is used to fit the change process of the remaining mass with time (Olson, 1963) (3).


(1) $$MR=(X_{t}/X_{0})\times 100\%$$

(2) $$E(\%)=\left[\left(C_{t}\times X_{t})/(C_{0}\times X_{0}\right)\right]\times 100\%$$

(3) $$X_{t}/X_{0}=ae^{-kt}$$

Here, *X_0_* (g) is the initial litter mass, *X_t_* (g) is the mass remaining after *t* years of decomposition, *C_0_* is the initial nutrient concentration, and *C_t_* is the nutrient concentration at time *t*, *k* is decomposition coefficient (Song et al., 2015). All statistical analyses of the data were performed using SPSS 24.0 software (SPSS Inc., Chicago, IL, USA). One-way ANOVA and Duncan’s method were used to analyze variance and multiple comparisons (*P* < 0.05 used as threshold), and the interactions among N addition, root order classes, and decomposition time were studied by three-way ANOVA. Pearson correlation analysis was used to explore the correlation between fine root decomposition coefficient, nutrient concentration and soil characteristics. Excel 2016 was used for data statistics and plotting.

## Results

### Initial substrate chemistry

The initial nutrient and C fraction concentrations were significantly (*P* < 0.05) different among the three root orders before their placement in the litterbags (**Table 1**). The initial N and P concentrations of the lower-order fine roots were significantly higher than those of higher-order fine roots (*P* < 0.05). The concentrations of cellulose, soluble sugar, and starch were significantly lower than those of the higher-order fine roots (*P* < 0.05, **Table 1**). No significant differences were found in the C and lignin concentrations. The contrasting concentrations of N and P among the three root orders also led to an opposing gradient in C/N, C/P, and lignin/N ratio, with significant (*P* < 0.05) differences among the root orders, with order 1–2 < order 3–4 < order 5–6 (**Table 1**).

**Table 1:** Initial chemistry of the fine roots in *Pinus massoniana*.

Root Class	Root C Fraction (％)				Root Nutriment (mg/g)			Ratios		
	Lignin	Cellulose	Soluble sugar	Starch	C	N	P	C/N	C/P	Lignin/N
Order 1–2	20.49 ± 0.61a	27.1 ± 1.28c	4.48 ± 0.11b	5.16 ± 0.26c	425.50 ± 6.88a	3.85 ± 0.29a	0.53 ± 0.02a	110.92 ± 6.68b	803.16 ± 12.03c	53.53 ± 5.38b
Order 3–4	20.59 ± 1.47a	32.06 ± 1.02b	5.52 ± 0.04a	6.89 ± 0.32a	433.20 ± 3.41a	2.86 ± 0.12b	0.39 ± 0.02b	151.85 ± 7.84a	1121.3 ± 56.02b	72.31 ± 7.91a
Order 5–6	22.58 ± 1.32a	34.92 ± 0.50a	5.58 ± 0.10a	6.31 ± 0.26b	436.92 ± 6.24a	2.72 ± 0.06b	0.29 ± 0.04c	160.5 ± 2.69a	1528.61 ± 194.97a	82.95 ± 5.00a

Values are means ± SE (n=3). Different letters indicate significant differences among the three order fine roots (P < 0.05).

### Fine root mass loss and decomposition rate

**Figure 1:**
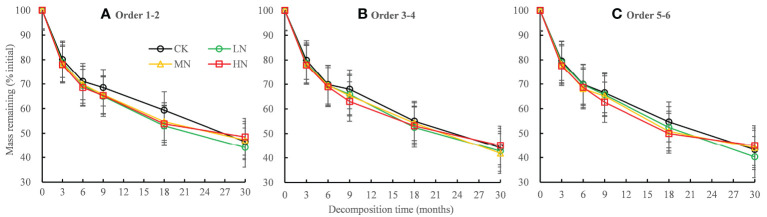
Residual percentages of the initial mass during the 30-month fine root decomposition of *Pinus massoniana* in different N addition treatments. Values are means ± SE. The different colors denote CK (0 kg ha^-1^ year^-1^), LN (30 kg ha^-1^ year^-1^), MN (60 kg ha^-1^ year^-1^), and HN (90 kg ha^-1^ year^-1^). **(A–C)** represent the mass remaining of order 1-2, order 3-4 and order 5-6 fine roots, respectively.

The mass remaining of fine roots decreased rapidly in the first 6 months of decomposition and decreased slowly in the 6−30 months of decomposition (**Figure 1**). The root data of the initial mass remaining in each treatment fitted well with the single exponential model (*R* ^2^ range 0.846–0.946; **Table 2**). The ANOVA showed that N addition, root order, decomposition time, and the interactions with each other have significant effects on mass remaining (mostly *P* < 0.001; **Table 3**). In the 0−18 months of decomposition, all N addition treatments significantly promoted the decomposition of fine roots (*P* < 0.05), and the average decomposition rates of order 1–2, order 3–4, order 5–6 fine roots were increased by 13.54%, 6.15% and 7.96% respectively. After 30 months of decomposition, low N treatment promoted the decomposition of fine roots in all orders, whereas high N addition inhibited the decomposition of fine roots, and the average decomposition rates of order 1–2, order 3–4, order 5–6 fine roots were decreased by 58.35%, 35.43% and 47.56% respectively. (**Figure 1**, **Table 2**). The lower-order fine roots (order 1–2) decomposed slower than the higher-order fine roots (order 3–4 and order 5–6). The average decomposition constant (*k*) of the order 1–2 fine roots (0.272) was less than that of the order 3–4 (0.300) and order 5–6 fine roots (0.307) (**Table 2**, *P* < 0.05).

**Table 2:** Mean decomposition rates (*k*) of the three orders of roots in different N addition treatments.

Order	Treatment	Decomposition period (a) and constant *k* (a^-1^)					
		0-1.5	*R^2^*	*P*	0-2.5	*R^2^*	*P*
1-2	CK	0.313 ± 0.037Bb	0.846	0.000	0.269 ± 0.02Cb	0.919	0.000
	LN	0.390 ± 0.035Aa	0.907	0.000	0.297 ± 0.023Ba	0.910	0.000
	MN	0.366 ± 0.037Ba	0.885	0.000	0.267 ± 0.024Bb	0.882	0.000
	HN	0.375 ± 0.039Ba	0.879	0.000	0.257 ± 0.027Bc	0.846	0.000
3-4	CK	0.362 ± 0.034Ab	0.898	0.000	0.295 ± 0.020Bb	0.930	0.000
	LN	0.394 ± 0.033Aa	0.914	0.000	0.309 ± 0.022Ba	0.926	0.000
	MN	0.371 ± 0.037Bab	0.885	0.000	0.311 ± 0.021Aa	0.932	0.000
	HN	0.388 ± 0.040Bab	0.881	0.000	0.284 ± 0.026Ab	0.880	0.000
5-6	CK	0.369 ± 0.035Ab	0.897	0.000	0.303 ± 0.021Ab	0.931	0.000
	LN	0.397 ± 0.033Aab	0.918	0.000	0.332 ± 0.020Aa	0.946	0.000
	MN	0.415 ± 0.036Aab	0.912	0.000	0.300 ± 0.026Ab	0.890	0.000
	HN	0.430 ± 0.037Aa	0.912	0.000	0.292 ± 0.029Ab	0.860	0.000

Values represent mean ± SE (n = 3). Different capital letters indicate significant differences among different root orders under the same N addition treatment. Different lowercase letters indicate significant differences among N addition treatments under the same root order (P < 0.05).

**Table 3:** Effects of N treatment, root order, decomposition time and their interaction on the residual rate of fine root decomposition.

Index	N Treatment	Order	Time	N*Time	Order*Time	N*Order	N*Order*Time	*R^2^*
Mass remaining	***	***	***	***	***	*	***	0.998
Lignin	***	***	***	**	***	ns	ns	0.978
Cellulose	ns	***	***	ns	ns	**	ns	0.958
Soluble sugar	ns	ns	***	ns	ns	ns	ns	0.982
Starch	ns	***	***	**	***	ns	ns	0.993
C	*	***	***	ns	***	ns	ns	0.970
N	**	***	***	*	***	*	ns	0.882
P	***	***	***	*	***	ns	*	0.879
C/N	*	***	***	ns	***	ns	ns	0.714
C/P	***	***	***	*	**	ns	ns	0.549
Lignin/N	ns	***	***	ns	**	ns	ns	0.914

*P < 0.05, ** P < 0.01, ***P < 0.001, ns, not significant

### Root nutrient dynamics during root decomposition

**Figure 2:**
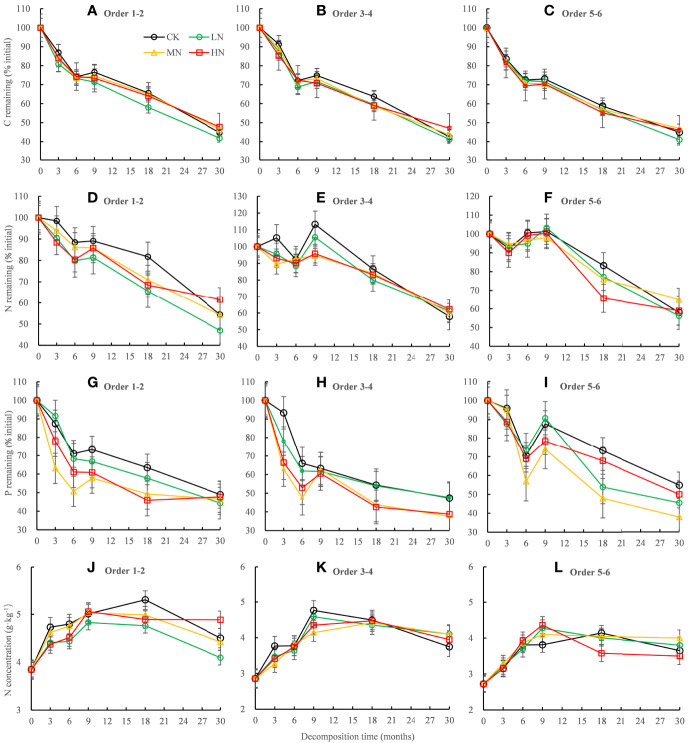
Residual percentages of C, N, and P and absolute concentrations of N during the 30-month fine root decomposition of *Pinus massoniana* in different N addition treatments. Values are means ± SE. The different colors denote CK (0 kg ha^-1^ year^-1^), LN (30 kg ha^-1^ year^-1^), MN (60 kg ha^-1^ year^-1^), and HN (90 kg ha^-1^ year^-1^). **(A–I)** represent the residual percentages of C, N, and P in order 1-2, order 3-4 and order 5-6 fine roots, respectively. **(J–L)** represent the absolute concentrations of N in order 1-2, order 3-4 and order 5-6 fine roots, respectively.

Residual C and P in all orders fine roots decreased rapidly in the first 6 months but increased slowly in the 6−9 months period and then decreased in the 9−30 months (**Figures 2A–C, G–I**). The variation trends of residual N in the different root orders were dissimilar. Residual N in the lower-order fine roots decreased with time (**Figure 2D**), while N fixation occurred in the higher-order fine roots in the first 9 months of decomposition, and then decreased in the late decomposition stage (**Figures 2E, F**). The ANOVA showed that N addition, root order, and decomposition time, and the interaction between root orders and time had significant effects on C, N, and P residue rates (mostly *P* < 0.001, **Table 3**). After 30 months of decomposition, LN treatment promoted the release of C, N, while HN treatment has opposite effects. All the N addition treatments promoted P release.

**Figure 3:**
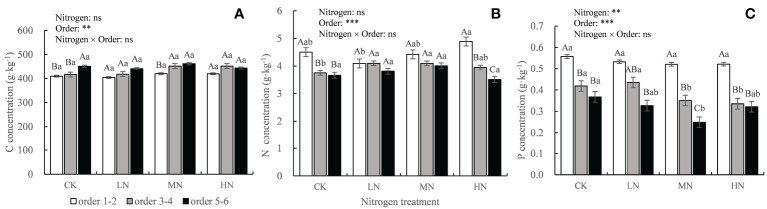
Absolute concentrations of C, N, and P in the decomposing roots in each treatment after 30 months of decomposition. Different capital letters indicate significant differences among different root orders under the same N addition treatment, and different lowercase letters indicate significant differences among the N addition treatments under the same root order (*P* < 0.05). **P < 0.01, ***P < 0.001, ns, no significant. CK: 0 kg ha^-1^ year^-1^; LN: 30 kg ha^-1^ year^-1^; MN: 60 kg ha^-1^ year^-1^; HN: 90 kg ha^-1^ year^-1^. **(A–C)** represent the absolute concentrations of C, N, and P in fine roots, respectively.

The N concentration in the fine roots increased continuously in the rapid stage of decomposition and then decreased gradually in the slow stage but was always higher than the initial N concentration (**Figures 2J–L**). After 30 months of decomposition, the absolute concentrations of N and P in the lower-order roots were significantly (*P* < 0.05) higher than those in the higher-order roots (**Figures 3B, C**), but C concentration was lower than that in the higher-order roots (**Figure 3A**). The two-way ANOVA showed that root order affected C, N, and P concentrations, while N addition only affected the final P concentration, resulting in a significant decrease (*P* < 0.01) (**Figure 3**).

### Root C fraction dynamics during root decomposition

**Figure 4:**
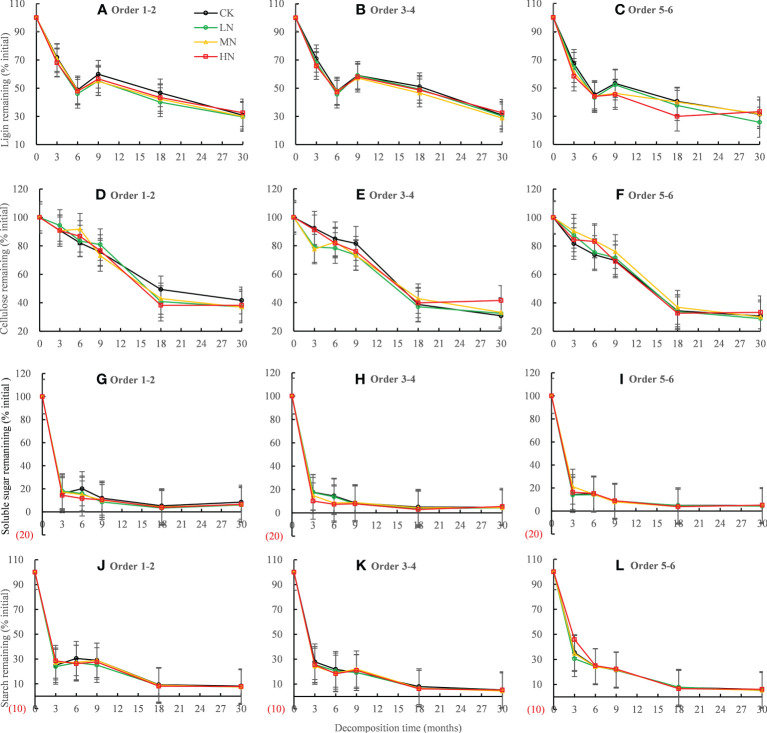
Residual percentages of lignin, cellulose, soluble sugar, and starch during the 30-month fine root decomposition of *Pinus massoniana* in different N addition treatments. Values are means ± SE. The different colors denote CK (0 kg ha^-1^ year^-1^), LN (30 kg ha^-1^ year^-1^), MN (60 kg ha^-1^ year^-1^), and HN (90 kg ha^-1^ year^-1^). **(A–L)** represent the lignin, cellulose, soluble sugar, and starch remaining in order 1-2, order 3-4 and order 5-6 fine roots, respectively.

Residual lignin decreased rapidly in the first 6 months of decomposition, increased in the following 6−9 months, and then decreased in 9−30 months (**Figures 4A–C**). Residual cellulose decreased rapidly by 50.61% ~ 67.33% in the first 18 months of decomposition, while it decreased very slowly in 18−30 months of decomposition (**Figures 4D–F**). The residue rates of soluble sugar and starch decreased rapidly in the first 3 months of decomposition, fluctuated in the following 3−18 months, and remained stable in the 18−30 months period (**Figures 4G–L**). The ANOVA showed that N treatment had a significant (*P* < 0.001) effect on lignin residue rate, but no significant effect on the cellulose, starch, and sucrose residue (**Table 3**). After 30 months of decomposition, LN treatment promoted lignin release, whereas HN treatment inhibited lignin release (**Figures 4A–C**).

### Soil properties and enzyme activities under N addition treatments

**Figure 5:**
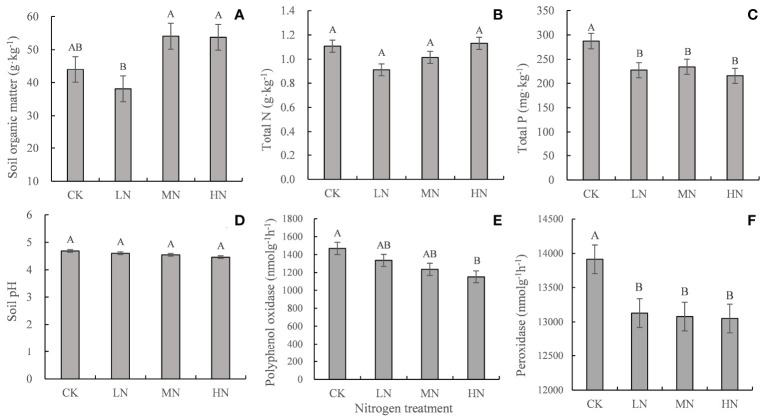
Soil properties and enzyme activities in the different treatments after 30 months of decomposition. Values represent the means ± SE (n = 3). Different capital letters indicate significant differences among different N addition treatments (*P* < 0.05). CK (0 kg ha^-1^ year^-1^), LN (30 kg ha^-1^ year^-1^), MN (60 kg ha^-1^ year^-1^), and HN (90 kg ha^-1^ year^-1^). **(A–C)** represent the content of soil organic matter, total N, total P, respectively. **(D–F)** represent the soil pH and the activity of polyphenol oxidase and peroxidase, respectively.

After 30 months of decomposition, LN treatment decreased the contents of soil organic matter and total N, whereas HN treatment had the opposite effect. All N treatments decreased soil pH, total P content, and polyphenol oxidase and peroxidase activities (**Figure 5**).

### Relationships between fine root decomposition and nutrient elements and soil characteristics

**Figure 6:**
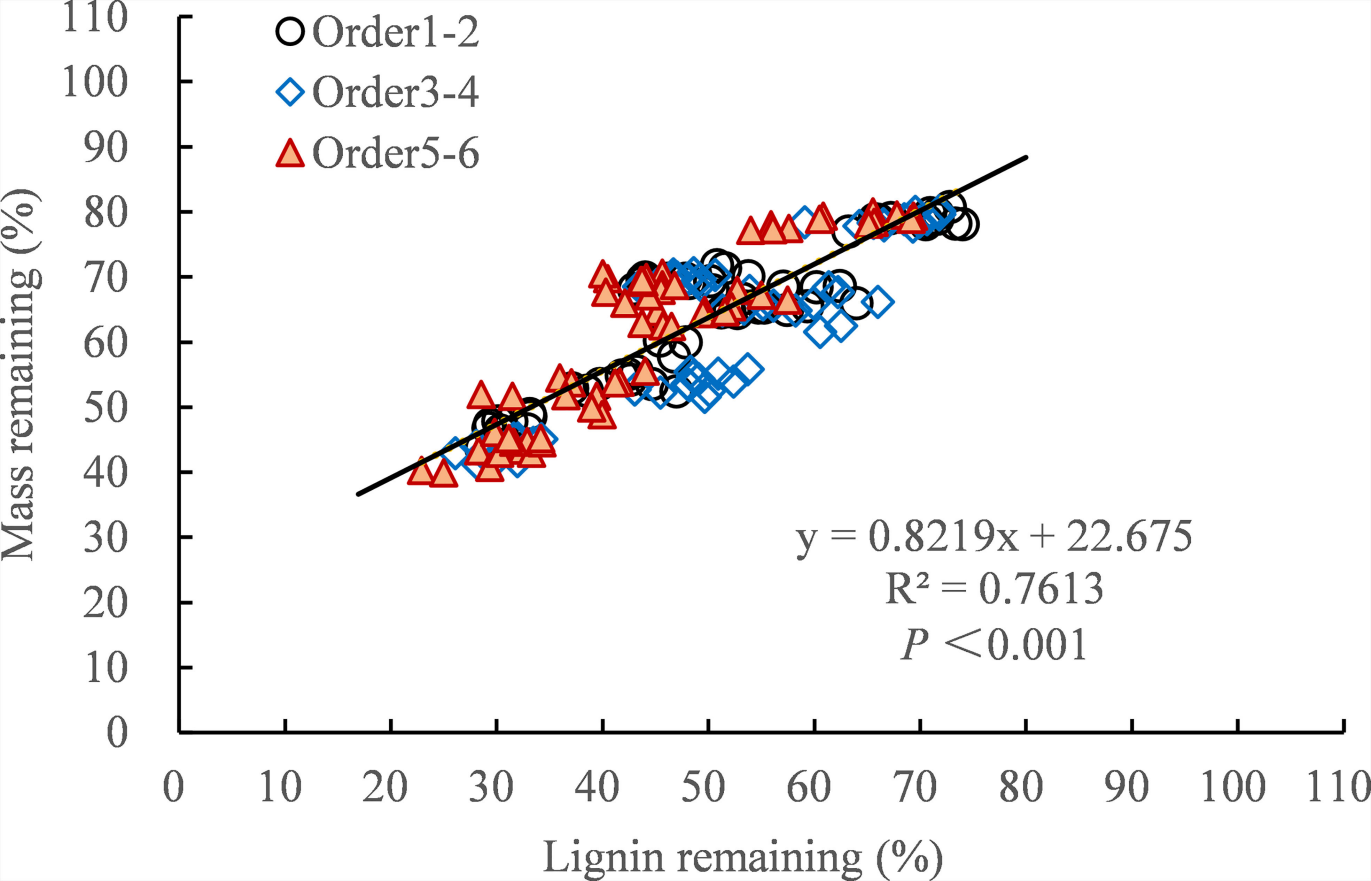
Relationship between residual mass and residual lignin after 30 months of decomposition.

After 30 months of decomposition, the decomposition constant (*k*) is closely related to the concentration of nutrient elements (**Table 4**). For example, the *k*-value was significantly positively correlated with the initial concentrations of cellulose, starch, soluble sugar, C and the ratio of C/N, C/P, and lignin/N (*P* < 0.01), and significantly negatively correlated with the initial and final concentration of N, P (*P* < 0.01). In addition, the *k*-value was also significantly positively correlated with soil peroxidase and polyphenol oxidase activities, and significantly negatively correlated with soil organic matter content (**Table 5**, *P* < 0.05). There was a significant positive linear correlation between residual mass and residual lignin and nitrogen during the 30-month decomposition process (**Figure 6**, *P* < 0.001).

**Table 4:** Correlation between the decomposition constant (*k*) and nutrient concentration of fine roots.

Index	Lignin	Cellulose	Soluble sugar	Starch	C	N	P	C:N	C:P	Lignin:N
Initial Concentration	0.597	.938^**^	.970^**^	.802^**^	.709^*^	-.943^**^	-.932^**^	.968^**^	.865^**^	.945^**^
Final Concentration	0.273	0.290	0.352	0.217	0.229	-.515^**^	-.552^**^	.492^**^	.450^**^	.431^**^

*P < 0.05, ** P < 0.01.

**Table 5:** Correlation between the decomposition constant (*k*) and soil characteristics.

Index	pH	Soil N	Soil P	Organic matter	Peroxidase	Polyphenol oxidase
*k*	0.480	-0.119	0.407	-.612^*^	0.331	0.545^*^

*P < 0.05.

## Discussion

### Effect of N addition on fine root decomposition

The main factors affecting the decomposition rate of the fine roots varied depending on the decomposition phase. The early decomposition stage was dominated by the degradation of soluble and relatively unstable compounds in the litter, including the initial total non-structural carbohydrates. The degradation of cellulose followed this, and finally, the acid-insoluble substances and lignin degradation became dominant (Berg and McClaugherty, 2008; Tu et al., 2014). In our study, two stages of decomposition with different mass-loss rates were observed. In the first 6 months of decomposition, the mass remaining of fine roots decreased rapidly with an average loss of 30.60% of the initial mass, whereas slower decomposition rates were observed in the following 6−30 months, with an average loss of 25.01% (**Figure 1**). Consistent with many previous studies (Yang et al., 2004; Berg and McClaugherty, 2008; Tu et al., 2015), we observed the degradation of starch and soluble sugar first, followed by a rapid decline in cellulose (**Figures 4D−L**). During 18−30 months of decomposition, the above nutrient residue rate remained unchanged, and the lignin residue rate continued to decline (**Figures 4A−C**), indicating that lignin decomposition was dominant in the late stage of decomposition.

Our first hypothesis was confirmed as we found that all concentrations of N addition promoted the decomposition rate of the fine roots in the early stage of decomposition (0−18 months), whereas HN treatment inhibited the degradation of the fine roots in the late stage of decomposition (18−30 months) (**Table 2**). The stimulating effect of N addition on the decomposition rates in the early stages may be partly explained by the altered soil enzyme activities that were involved in polysaccharide breakdown due to increased N inputs (Sun et al., 2016). Our previous research showed that N addition increased the activities of hydrolases (β-1-4 glucosidase, N-acetylglucosaminosidase, and acid phosphatase) and oxidase (polyphenol oxidase and peroxidase) after 9 months of decomposition (Wang et al., 2021). The increase in hydrolase and oxidase activities promoted soluble sugar, cellulose, and hemicellulose degradation in the early decomposition stage (Li et al., 2011; Sun et al., 2016; Dong et al., 2019). As our study found that nitrogen addition promoted the release of C, N, P, and lignin in the early decomposition stage (**Figures 2**, **4**). These results were largely consistent with those of studies showing that exogenous nitrogen stimulated the initial decomposition rate in the N-fertilizer experiment (Hobbie et al., 2012; Sun et al., 2016; Gill et al., 2021). With the progress of fine root decomposition, lignin decomposition became dominant in the late stage of decomposition (Berg and McClaugherty, 2008; Tu et al., 2015). There was a significant positive linear correlation between residual lignin and residual mass, which explained 76.13% of the variation in the residual mass (**Figure 6**). We observed that HN treatment inhibited the degradation of lignin (**Figures 4A−C**), which may be the main reason for the slow decomposition of fine roots caused by nitrogen addition. The inhibition of nitrogen addition on lignin decomposition may be related to the following mechanisms.

Firstly, a high-N environment may inhibit the synthesis of lignin-decomposing enzymes. The range of soil pH was 4.45−4.69 after 30 months of N addition (**Figure 5D**). However, the optimum pH ranges for the growth of Basidiomycetes and Ascomycetes related to lignin decomposition are 4.0−5.0 and 6.0−7.5, respectively (Sinsabaugh, 2010). Therefore, Basidiomycetes might be the leading lignin-degrading fungal community in this ecosystem. A previous study reported that Basidiomycetes do not synthesize lignin-degrading enzymes in the presence of ammonium or other low molecular weight N-rich compounds such as amino acids (Keyser et al., 1978). In addition, we found that N addition inhibited the activities of peroxidase and polyphenol oxidase related to the lignin degradation in the soil after 30 months of nitrogen addition experiment (**Figure 5**). As a matter of fact, many previous studies have found that N additions inhibited the activities of lignin-degrading enzymes in the later stages of fine root decomposition (Manzoni et al., 2010; Tu et al., 2014; Xia et al., 2018; Dong et al., 2022). Secondly, the added N reacts with the secondary compounds (mostly polyphenols) produced during lignin decomposition and is fixed into the fine roots, resulting in the continuous accumulation of anti-decomposition substances and N in fine roots, which further slows down the decomposition rate of the fine roots (Hansen et al., 2009; Hobbie et al., 2012). In the present study, the higher residual N (46.90% ~ 65.90%) in the fine roots observed after 30 months of decomposition may be due to the interaction between exogenous inorganic N addition and residual lignin (**Figures 2D−F**). Some studies have found that exogenous inorganic N ions will be adsorbed by acidic non hydrolyzable residues in the substrates to form new insoluble substances, and directly polymerize with lignin to produce more refractory humus, thus inhibiting fine root decomposition (Preston et al., 2009; Berg et al., 2013; Kou et al., 2018). By contrast, other studies showed that nitrogen addition had no significant effect on the activity of lignin decomposing enzymes in litter layer or soil (Hobbie, 2008; Keeler et al., 2009). In addition, some studies have also found that the effect of nitrogen addition on oxidase activity depends on the availability of nitrogen in the environment, and even get opposite results in different forest types. For example, a study found that the activity of polyphenol oxidase decreased in the black oak-white oak mixed forest but increased in the mixed forest of sugar maple-red oak and sugar maple-basswood mixed forest after one year of nitrogen application (Waldrop et al., 2004). Therefore, the mechanism of inhibition of N addition on late-stage decomposition may vary depending on specific litter types and/or forest sites (Keeler et al., 2009).

In general, the distribution of C from plant to soil is mainly completed through plant fine roots in forest ecosystems (Van Groenigen et al., 2006; Song et al., 2016). C input primarily exists in the form of active rhizosphere sediments (Boddy et al., 2007). Recent findings show that the transformation of root-derived matters into stable soil organic matter components is greater than that of leaf litter (Bird et al., 2008; Mambelli et al., 2011). Therefore, the variation of root decomposition may affect the accumulation of soil organic matter in forest ecosystem. Tu et al. (2015) found that the later stage of root decomposition (the stage when mass loss tends to be stable) is mainly the stage of humus formation. N addition may promote the accumulation of stable C components in the soil by inhibiting the later stages of fine root decomposition. After 30 months of N addition, we observed that low N treatment promoted fine root decomposition and decreased the soil organic matter content, whereas high N inhibited fine root decomposition and increased soil organic matter content (**Figure 5A**). This is consistent with the previous conclusion that low nitrogen (30 kg N ha^−1^ year^−1^) reduces soil C storage and high nitrogen (≥90 kg N ha^−1^ year^−1^) has the opposite effect (Song et al., 2016). In fact, many previous studies have shown that the increase of soil organic matter is generally related to the slower decomposition of plant residues rather than the more significant litter input (Zak et al., 2008; Janssens et al., 2010; Xia et al., 2018). N addition can increase soil carbon storage by inhibiting heterotrophic respiration, reducing microbial biomass and altering microbial community composition and carbon degrading enzyme activity (Xia et al., 2018; Dong et al., 2022). Correlation analysis showed that soil organic matter content was significantly negatively correlated with fine roots decomposition coefficient (*k*) (**Table 4**). Therefore, we suggested that the inhibition of N addition on the late decomposition stage of fine roots may partly explain the increase of soil organic matter under high N deposition. In addition, a previous study showed that long-term simulated N deposition accelerated soil acidification (Yu et al., 2021). Our previous studies found that the soil pH and total P content decreased after 9 months of N addition (Wang et al., 2021). This phenomenon recurred after 30 months of N addition, indicating that increasing N deposition will lead to soil acidification. Due to the low solubility of P compounds at low pH, N deposition-induced acidification may limit the release of P radicals (Chapin et al., 2011). Furthermore, N inputs can enhance phosphatase production and consequently cause some ecosystems to shift from relative N limitation to P limitation (Marklein and Houlton, 2012). Our study also found that N addition enhanced acid phosphatase activity (**Figure S1**), indicating that the soil needs more enzyme hydrolysis to obtain the required phosphorus.

### Differences in fine root decomposition in the different order classes

Studies have shown significant differences in the physiological functions of different root orders (Guo et al., 2004; Kou et al., 2015). For example, the higher-order roots mainly perform the functions of transportation, storage, and structural support, while the lower-order roots are used primarily to obtain nutrients and absorb water. These differences in root function are reflected in the histochemical composition and decomposition rate (Fan and Guo, 2010; Wang et al., 2014; McCormack et al., 2015).

Our second hypothesis was confirmed as we found that the decomposition rate of the higher-order roots was faster than that of the lower-order roots during the 30−month decomposition (**Table 1**, **Figure 1**). This is consistent with many previous conclusions (Sun et al., 2013; Yang et al., 2019). The mycorrhizal hypothesis suggests that EM mycorrhizal fungi are more likely to infect lower-order fine roots during decomposition, and mycorrhizal sheaths are rich in chitin. The high chitin content may reduce the decomposition of fine roots, resulting in slower decomposition of the lower-order fine roots than the higher-order fine roots (Langley et al., 2003a; Langley and Hungate 2003b; Guo et al., 2008). In addition, a previous study has shown that microbial decomposition will slow down due to the limitation of carbon quality (Moorhead and Sinsabaugh, 2006). The low decomposition rate of the lower-order fine roots may be due to the combined effect of high nitrogen concentration and low carbon concentration (Yang et al., 2019). In our study, we observed that the higher-order roots contained higher concentrations of initial cellulose and soluble sugars, starch, C and higher C/N, C/P, and lignin/N ratios (**Table 1**). Correlation analysis showed that the decomposition constant (*k*) was significantly positively correlated with the above indicators, and significantly negatively correlated with the initial and final N and P concentrations (**Table 4**). After 30 months of decomposition, the absolute concentrations of N and P in the higher-order roots were significantly slower than those in the lower-order roots (**Figures 3B, C**). Therefore, the higher-order fine roots decomposed faster than the lower-order fine roots due to higher initial concentrations of cellulose, starch, soluble sugars, C and higher C/N, C/P, and lignin/N ratios and lower N and P concentrations. Same as our conclusion, a 23-month decomposition experiment also found that the litter decomposition rate was controlled strongly by the initial nutrient content and C/N, C/P ratios (Zhang et al., 2021).

Previous research showed that the global reserves of litter estimated by the short-term decomposition coefficient is underestimated by at least one-third (Harmon et al., 2009; Moore et al., 2017). Our study observed that the fine root system retained a considerable part of the initial mass (40.29%~ 48.46%) after 30 months of decomposition. In the first 18 months of this study, the root decomposition coefficient was significantly greater than that in the entire decomposition stage (**Table 2**), indicating that the short-term root decomposition coefficient would overestimate the root decomposition rate. These studies show that root decomposition is a long-term process and long-term work is needed to fully understand the effects of N deposition on decomposition.

## Conclusion

The effect of nitrogen addition on decomposition varied with decomposition stages. N addition promotes early-stage and inhibits late-stage decomposition of fine roots. The inhibition of lignin-degrading enzyme activity by N addition may be the mechanism of the observed negative effect on the late decomposition stage. The decomposition difference between the lower-order and higher-order fine roots were controlled strongly by the initial chemical quality of the fine roots. High N addition increased soil organic matter content and decreased soil pH and P content, indicating that increasing N deposition would increase soil C sequestration, but also lead to soil acidification in *Pinus massoniana* plantations. This study provides new insights into understanding and predicting possible changes in plant root decomposition and soil properties in the future atmospheric N deposition increase scenarios. However, due to the fine root system retained a considerable part of the initial mass (40.29%~ 48.46%) after 30 months of decomposition, short-term root decomposition coefficient would overestimate the root decomposition rate. Thus, long-term work is needed to fully understand the effects of N deposition on decomposition.

## Data availability statement

The original contributions presented in the study are included in the article/**Supplementary Material**. Further inquiries can be directed to the corresponding author.

## Author contributions

LW and RC designed the experiments and wrote the original draft manuscript. YS, PS, TC, and MZ conducted the field trial and the laboratory analysis. RC, LZ, and WX critically reviewed and edited the preliminary draft. All authors contributed to the article and approved the submitted version.

## Funding

This study was supported by the Fundamental Research Fund of the Chinese Academy of Forestry (No. CAFYBB2021ZE003) and the National Key Research and Development Program of Ministry of Science and Technology of China (No. 2016YFD0600204).

## Acknowledgments

We thank the National Forest Ecosystem Station of Three Gorges Reservoir in Zigui County for their support in our field work. We also thank Editage (www.editage.cn) for English language editing.

## Conflict of interest

The authors declare that the research was conducted in the absence of any commercial or financial relationships that could be construed as a potential conflict of interest.

## Publisher’s note

All claims expressed in this article are solely those of the authors and do not necessarily represent those of their affiliated organizations, or those of the publisher, the editors and the reviewers. Any product that may be evaluated in this article, or claim that may be made by its manufacturer, is not guaranteed or endorsed by the publisher.
