# Supplementary material for: Nitrogen addition promotes early-stage and inhibits late-stage decomposition of fine roots in Pinus massoniana plantation
Source: Front Plant Sci. 2022 Nov 14;13:1048153. doi: 10.3389/fpls.2022.1048153 (PMC9701838; doi:10.3389/fpls.2022.1048153)
Supplement: Supplementary file 1 [file DataSheet_1.docx]

**Supplementary Material**

**Nitrogen addition promotes early-stage and inhibits late-stage decomposition of fine roots in *Pinus massoniana* plantation**

Lijun Wang^1^, Yafei Shen^1,2^, Ruimei Cheng^1,2*^, Wenfa Xiao^1,2^, Lixiong Zeng^1,2^, Pengfei Sun^1^, Tian Chen^1^, Meng Zhang^1^

^1^ Ecology and Nature Conservation Institute, Chinese Academy of Forestry, Key Laboratory of Forest Ecology and Environment, National Forestry and Grassland Administration, Beijing 100091, China

^2^ Co-Innovation Center for Sustainable Forestry in Southern China, Nanjing Forestry University, Nanjing 210037, China

* Correspondence: Ruimei Cheng E-mail address: [cafcheng@sina.com](mailto:cafcheng@sina.com)


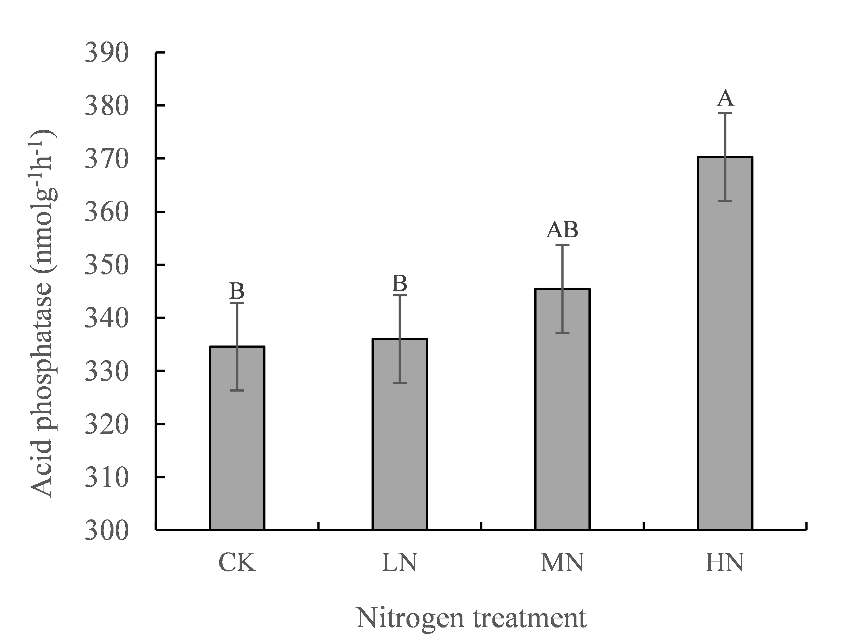


**FIGURE S1** Acid phosphatase activity in the different treatments after 30 months of N addition. Values represent the means ± SE (n = 3). Different capital letters indicate significant differences among different N addition treatments (*P* < 0.05). CK (0 kg ha^-1^ year^-1^), LN (30 kg ha^-1^ year^-1^), MN (60 kg ha^-1^ year^-1^), and HN (90 kg ha^-1^ year^-1^).
